# Novel Bacterial Surface Display System Based on the *Escherichia coli* Protein MipA

**DOI:** 10.4014/jmb.2001.01053

**Published:** 2020-04-23

**Authors:** Mee-Jung Han

**Affiliations:** Department of Biomolecular and Chemical Engineering, and Department of Nursing, Dongyang University, Yeongju 36040, Republic of Korea

**Keywords:** *E. coli* MipA, cell surface display, outer membrane protein

## Abstract

Bacterial surface display systems have been developed for various applications in biotechnology and industry. Particularly, the discovery and design of anchoring motifs is highly important for the successful display of a target protein or peptide on the surface of bacteria. In this study, an efficient display system on *Escherichia coli* was developed using novel anchoring motifs designed from the *E. coli mipA* gene. Using the C-terminal fusion system of an industrial enzyme, *Pseudomonas fluorescens* lipase, six possible fusion sites, V^140^, V^176^, K^179^, V^226^, V^232^, and K^234^, which were truncated from the C-terminal end of the *mipA* gene (MV^140^, MV^176^, MV^179^, MV^226^, MV^232^, and MV^234^) were examined. The whole-cell lipase activities showed that MV^140^ was the best among the six anchoring motifs. Furthermore, the lipase activity obtained using MV^140^ as the anchoring motif was approximately 20-fold higher than that of the previous anchoring motifs FadL and OprF but slightly higher than that of YiaTR232. Western blotting and confocal microscopy further confirmed the localization of the fusion lipase displayed on the *E. coli* surface using the truncated MV^140^. Additionally the MV^140^ motif could be used for successfully displaying another industrial enzyme, α-amylase from *Bacillus subtilis*. These results showed that the fusion proteins using the MV^140^ motif had notably high enzyme activities and did not exert any adverse effects on either cell growth or outer membrane integrity. Thus, this study shows that MipA can be used as a novel anchoring motif for more efficient bacterial surface display in the biotechnological and industrial fields.

## Introduction

Bacterial surface display is a protein engineering technique used for display of a target, such as a peptide or protein (enzyme) on the surface of bacteria. Bacterial cell surface display systems have been employed for various biotechnological and industrial applications, such as whole-cell biocatalysts, biosensors, bioabsorbents and affinity-based screening, antibody epitope mapping, and vaccine delivery [1-3]. In particular, *Escherichia coli* display systems have been widely used as bioabsorbents and biosensors in the bioremediation of pollutants and toxic materials [4, 5], or as biocatalysts in biofuel and chemical production by the enzymatic transformation of chemicals to produce enantiomerically pure compounds [6-9].

However, the *E. coli* display system encounters difficulty in displaying a large protein stably and with the correct steric conformation because the protein must pass through two membrane layers and then undergo protein targeting. Thus, the choice and design of anchoring motifs are highly important for the stability of cell envelope integrity. Various anchoring motifs, including outer membrane proteins, lipoproteins, autotransporters, subunits of surface appendages, and S-layer proteins, were examined [1, 10, 11]. Most importantly, bacterial outer membrane proteins, such as FadL, LamB, Lpp-OmpA, OmpA, OmpC, OmpF, OmpS, OmpX, OmpW, OprF, PhoE, and YiaT, have been employed for displaying various peptides and proteins, including antibodies, domains, enzymes, and receptors [12-16]. However, each anchoring motif has been found to have different capacities for protein display, making it necessary to develop appropriate anchoring motifs depending on individual proteins of various sizes and characteristics [1, 17]. The majority of the previously developed systems are only suitable for peptides or relatively small polypeptides [1, 18]. Larger proteins could be displayed as anchoring motifs, such as *Pseudomonas aeruginosa* OprF [9], *E. coli* FadL [8], OmpX [19] and YiaT [20]. Therefore, the success of a cell surface display system is highly dependent on the choice of an anchoring motif that is appropriate for the intended target protein.

In this study, we developed an efficient *E. coli* cell surface display using a novel anchoring motif truncated from the *E. coli* MltA-interacting protein (MipA; Swiss-Prot no. P0A908) at the C-terminus. To determine the best anchoring motif from MipA, several possible motifs were tested by creating truncated *mipA* genes to link the function of a protein, specifically a highly thermostable lipase from *Pseudomonas fluorescens* SIK W1 (49.9 kDa) using a C-terminus deletion strategy. Additionally, the display efficiency of lipase using the truncated MipA motif was compared with those of the previous anchoring motifs, FadL [8], OrpF [9], and YiaT [20]. Furthermore, the best truncated MipA motif was employed to display another enzyme, α-amylase from *Bacillus subtilis* (47.3 kDa), which hydrolyzes large α-linked polysaccharides, such as starch or glycogen, into fermentable sugars.

## Materials and Methods

### Bacterial Strains and Culture Conditions

Table 1 shows all bacterial strains and plasmids employed in this study. *E. coli* XL-1 Blue was used as the host strain for general cloning, while *E. coli* XL10-Gold was used as the host strain for the cell surface display studies. *E. coli* cells were cultivated at 37°C and 250 rpm in a 250-ml flask containing 100 ml of Luria-Bertani (LB) medium composed of 10 g/l bacto-tryptone, 5 g/l bacto-yeast extract, and 5 g/l NaCl. For cultivation of recombinant *E. coli* cells harboring a plasmid, the medium was supplemented with ampicillin (50 μg/ml). Cell growth was monitored by measuring the optical density at 600 nm (OD_600_) using a spectrophotometer (Beckman DU 650, USA). At an OD_600_ of 0.4, the cells were induced to display lipase as a target protein by the addition of 1 mM isopropyl-β-D- thiogalactopyranoside (IPTG). After induction, the cells were cultured for an additional 4 h at 30°C and then used for further analyses.

### DNA Manipulation

Table 2 shows the primers used in this study. Polymerase chain reaction (PCR) to amplify target genes described in Table 2 was performed with a PCR Thermal Cycler MP (Takara Shuzo Co., Ltd., Japan) using the Expand High Fidelity PCR System (Roche Molecular Biochemicals, Germany). DNA sequencing was carried out using the BigDye Terminator Cycle Sequencing Kit (Perkin-Elmer Co., USA) with *Taq* polymerase and an ABI Prism 377 DNA sequencer (Perkin-Elmer Co.). All DNA manipulations, including digestion of restriction enzymes, ligation, and agarose gel electrophoresis, were performed according to standard procedures [21].

### SDS-PAGE and Immunoblotting

To confirm an enzyme display on the *E. coli* surface, the outer membrane proteins were prepared by sodium lauryl sarcosinate (sarcosine) enrichment, as previously described by Lee *et al*. [8, 9]. The outer membrane fractions were analyzed by 10% sodium dodecyl sulfate-polyacrylamide gel electrophoresis (SDS-PAGE). An immunoblotting experiment was performed in our previous study [22]. The proteins were transferred to Immobilon-P PVDF membranes (Millipore); the membranes were stained with MemCode reversible protein stain (Pierce Biotechnology) and imaged to verify that the protein loads were uniform and to ensure that efficient electrotransfer occurred, and the membranes were destained with Milli-Q water and blocked with nonfat dry milk prior to incubation with each primary antibody. For the immunodetection of the fusion protein, a monoclonal ANTI-FLAG M2 antibody (Sigma-Aldrich Co., USA) and a goat anti-mouse immunoglobulin G (IgG)- horseradish peroxidase (HRP) conjugate (Sigma-Aldrich) were used. An enhanced chemiluminescence (ECL) kit (Amersham ECL Prime Western Blotting Detection Reagent; GE Healthcare Bio-Sciences AB, Sweden) was used for signal detection.

### Immunofluorescence Microscopy

For fluorescence imaging, cells induced with 1 mM IPTG for 4 h were harvested by centrifugation for 5 min at 3,500 ×*g* and 4°C and then washed with phosphate-buffered saline (PBS). The cells were incubated with the ANTI-FLAG M2 antibody conjugated with fluorescein isothiocyanate (FITC) (Sigma-Aldrich) diluted 1:500 in PBS containing 3% (wt/vol) BSA at 25°C for 2 h. Prior to microscopic observation, the cells were washed three times with PBS to remove unbound antibody probes. The cells were mounted on poly-L-lysine–coated microscopic slide glasses and examined by confocal microscopy (Carl Zeiss, Germany). Photographs were taken with a Carl Zeiss LSM 410. The samples were excited at 488 nm, and the images were filtered by a longpass 505-nm filter.

### Measurement of Enzymatic Activities

The activity of the *P. fluorescens* lipase was determined by a spectrophotometric method using *p*-nitrophenyl decanoate (MW 293.36) as the substrate, as previously described by Lee *et al.* [8,9]. After cultivation, cells were harvested by centrifugation at 3,500 ×*g* and 4°C and then washed twice with PBS. Lyophilized cells were added to 3 ml of a substrate solution with a volumetric ratio of 1 part 10 mM *p*-nitrophenyl decanoate in acetonitrile, 4 parts ethanol and 95 parts 50 mM Tris-HCl. The reaction mixture was incubated at 37°C for 10 min, and the reaction was terminated by the addition of 2 μl of 0.5 M EDTA. The activity was determined by measuring the absorbance at 405 nm using a spectrophotometer. One unit of lipase activity was defined as the amount of enzyme releasing 1 μmol of *p*-nitrophenol per minute.

The activity of *B. subtilis* α-amylase was measured using an EnzyChrom α-Amylase Assay Kit (ECAM-100; BioAssay Systems, USA). Cells induced with 1 mM IPTG for 4 h were harvested by centrifugation for 5 min at 3,500 ×*g* and 4°C and then washed with MilliQ water, and transferred to a clear, flat-bottomed 96-well plate. The first reaction was carried out using starch as a substrate at room temperature (RT) for 15 min, followed by adding the detection reagent to each 96-well plate and incubation for 20 min at RT. Sample background readings were measured with an assay buffer (pH 7.0) at 585 nm, and 400 μM glucose was used as a standard. The α-amylase activity is calculated as:


$${\text{Activity = [(OD}}_{\text{Sample}}-{\text{OD}}_{\text{Buffer}}\text{) }\times 400\times n]/\left[\left({\text{OD}}_{\text{STD}}-{\text{OD}}_{\text{Buffer}}\right)\times t(\min)\right]\left(U/L\right)$$

In this formula, OD_Sample_, OD_STD_, and OD_Buffer_ are the optical density values of the sample, the 400 μM glucose standard and assay buffer, respectively, t is the incubation time, and n is the dilution factor. One unit (U) was defined as the amount of enzyme required to produce 1 μmol of glucose per minute under the assay conditions.

All activity assays were independently performed in triplicate, and the standard deviations were determined.

## Results and Discussion

### Design of the *E. coli* Surface Display System Using an *mipA* Gene

The function of *E. coli* MipA has not been well characterized to date, except that MipA may be implicated in antibiotic resistance in the *E. coli* outer membrane [23]. By BLAST analysis, MipA and OmpV were determined to be highly homologous and to belong to the MipA/OmpV family. However, our previous study of the outer membrane proteome showed that MipA was continually expressed in both *E. coli* K-12 and B strains [24]. Additionally, other researchers identified MipA by proteome analysis of the *E. coli* outer membrane [25,26]. Thus, MipA has been considered to engineer an outer membrane anchoring element for bacterial cell surface display due to its continuous expression independent of *E. coli* strains.

**Fig. 1:**
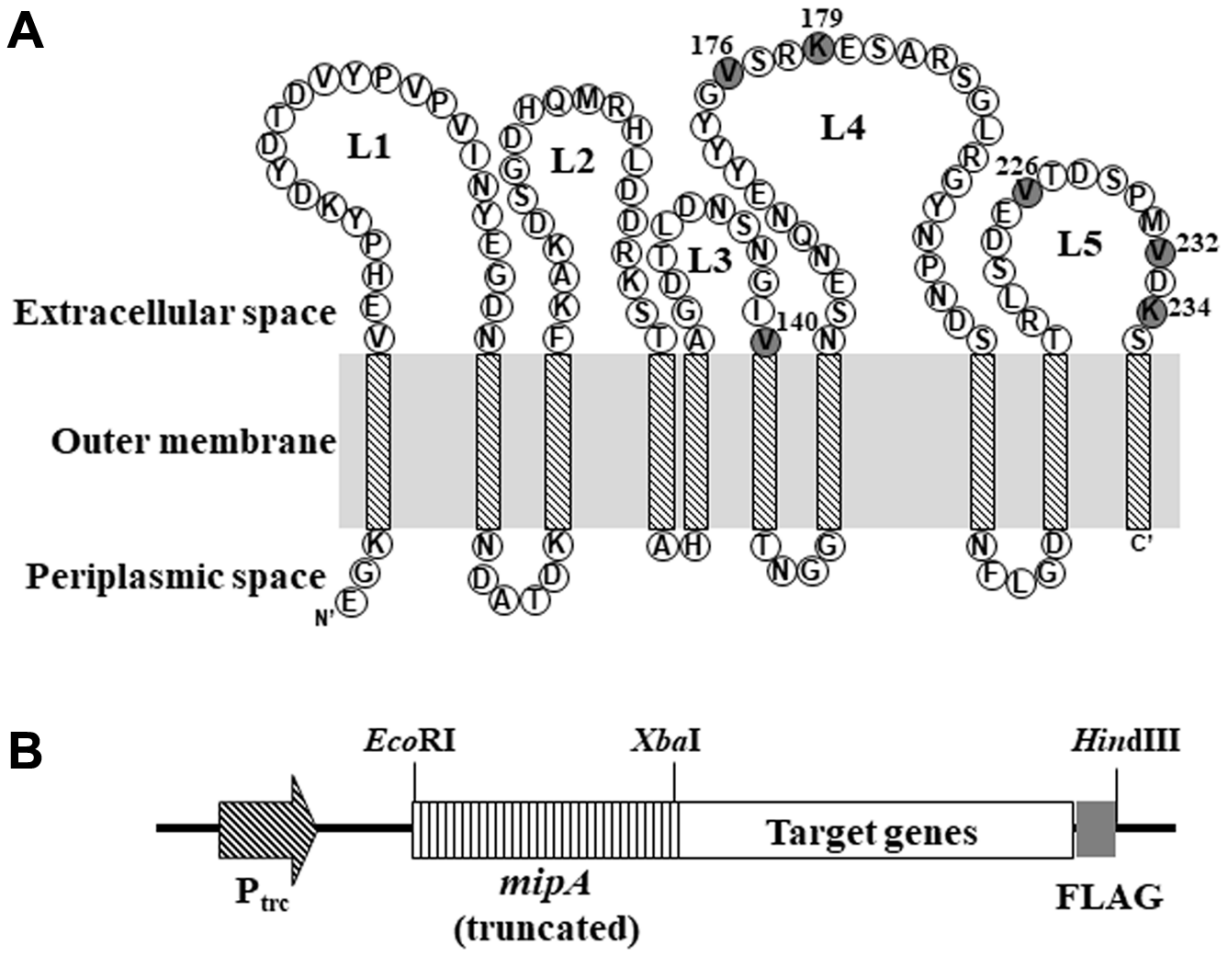
Construction of the MipA-fused display system. (**A**) The structure of MipA in the *E. coli* outer membrane. This topology was an arbitrary redrawing of the image predicted by PRED-TMBB [27]. Each gray circle indicates the fusion position of MipA with lipase: V^140^ in the third extracellular loop; V^176^ and K^179^ in the fourth extracellular loop; and V^226^, V^232^, and K^234^ in the fifth extracellular loop. (B) A schematic diagram of the expression system for MipA-fused target genes.

The outer membrane topology of MipA was first predicted using PRED-TMBB (http://bioinformatics.biol.uoa.gr/PRED-TMBB/ ) [27]. As shown in Fig. 1A, the MipA protein contains five extracellular loops that form a β- sheet protruding from the cell surface. Among these loops, the third, fourth and fifth loops were primarily considered, since they likely have stronger and more stable anchoring locations in the β-barrel structure of *E. coli*. In this study, a C-terminal truncation strategy was used to display the protein of interest, and six cleavage sites (V^140^, V^176^, K^179^, V^226^, V^232^, and K^234^) of the *mipA* gene were tested as possible fusion sites from loops 3, 4 and 5 exposed on the exterior of the outer membrane. *P. fluorescens* SIK W1 lipase (49.9 kDa) was examined as a model protein for display on bacterial surfaces due to its various applications in biocatalysis and bioremediation [5, 9, 13, 14].

### Construction of the Lipase Display System on the *E. coli* Surface

For construction of expression systems composed of truncated MipA fused to a target protein (Fig. 1B), the full- length *mipA* gene, as well as the C-terminal truncated *mipA* (*mipAt*) genes encoding the first 140, 176, 179, 226, 232 and 234 amino acids from the N-terminus, were amplified by PCR using the primers shown in Table 2. The genes were cloned into the *Eco*RI and *Xba*I sites of pTrc99A to make pTrcM, pTrcMV^140^, pTrcMV^176^, pTrcMK^179^, pTrcMV^226^, pTrcMV^232^, and pTrcMK^234^, respectively. To create a restriction enzyme site (XbaI) at the 3’ end of the *mipAt* gene, two amino acids (Ser and Arg) were added at the C-terminus. The full-length *mipA* gene without fusion (pTrcM) was used as a control.

To display a lipase on the *E. coli* cell surface, the *P. fluorescens* lipase gene containing the FLAG sequence (DYKDDDDK) was amplified using primers 15 and 16; it was then cloned into the XbaI and HindIII sites of the pTrcMV^140^, pTrcMV^176^, pTrcMK^179^, pTrcMV^226^, pTrcMV^232^, and pTrcMK^234^ vectors to create pTrcMV^140^PL, pTrcMV^176^PL, pTrcMK^179^PL, pTrcMV^226^PL, pTrcMV^232^PL, and pTrcMK^234^PL, respectively. *E. coli* XL10-Gold was used as a host strain for display because XL10-Gold was the best *E. coli* host strain, as reported by several previous display studies [8, 9, 20].

### Lipase Activity on the *E. coli* Surface

**Fig. 2:**
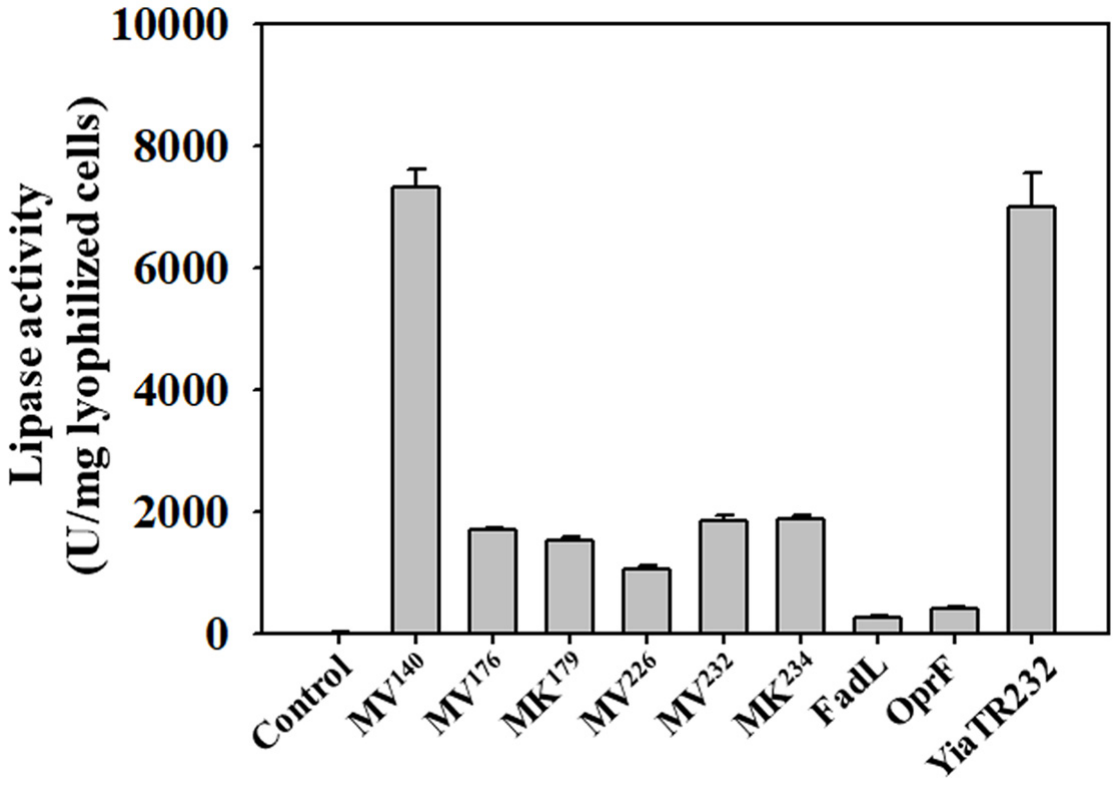
Comparison of lipase activity between the MipA anchoring motifs developed in this study and the previously reported motifs FadL [8], OprF [9], and YiaT [20]. *E. coli* XL10-Gold (pTrcM) is indicated as a control. All activity assays were independently performed in triplicate, and the standard deviations were determined. One unit (U) of lipase activity was defined as the amount of enzyme that releases 1 μmol of *p*-nitrophenol per minute.

To test which anchoring motif of the *mipA*-truncated derivatives developed in this study is the most efficient display system for displaying a large protein on *E. coli* cells, we first examined the enzymatic activities of the lipase displayed on several recombinant *E. coli* cells. The specific whole-cell lipase activities of recombinant cells are shown in Fig. 2. The results showed that lipase activity could be measured from all recombinant *E. coli* display strains to varying degrees, except for a control. Among the six *mipA* derivatives, MV^140^ was the best display motif. These results suggest that lipase was successfully and efficiently displayed in an active form with high stability using the *mipAt* gene as an anchoring motif. In addition, lipase activity obtained using MV^140^ was approximately 20-fold higher than those obtained with the previous anchoring motifs OprF or FadL display systems, which were applied as enantioselective biocatalysts for organic synthesis [8,9]. Furthermore, the display efficiency of the MV^140^-fused lipase on the membrane of *E. coli* had a slightly better efficiency than that of the YiaTR232-fused lipase [20], which was one of the more efficient *E. coli* display systems. These results suggest that the anchoring motif created using truncated MipA provides another efficient way to display functional lipase on the surface of *E. coli*.

### Confirmation of Lipase Display on the *E. coli* Surface

**Fig. 3:**
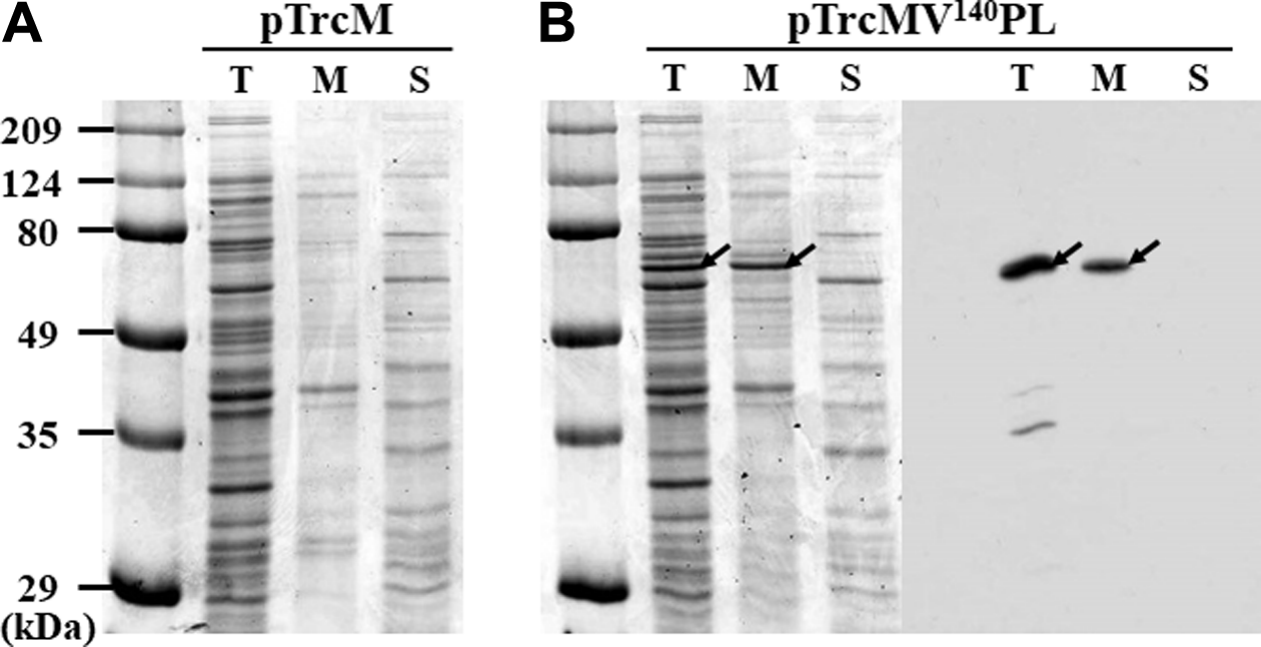
SDS-PAGE and western blotting analyses for total, outer membrane, and soluble fractions from *E. coli* XL10-Gold harboring pTrcM (control) (A) and pTrcMV^140^PL (B). First lane in each gel indicates the molecular weight size markers (kDa). T, whole cell lysate; M, outer membrane protein fractions; and S, soluble proteins. Arrows indicate the MV^140^-fused lipase.

To confirm that localization of lipase was displayed on the surface using MV^140^ as an anchoring motif, the total lysate, outer membrane proteins, and soluble protein fractions of the *E. coli* XL 10-Gold cells harboring pTrcMV^140^PL were analyzed by SDS-PAGE and western blotting (Fig. 3). No signal was detected in the *E. coli* cells harboring the control pTrcM. In contrast, the bands for the approximately 65-kDa fusion lipase proteins were clearly detected in the total lysate and outer membrane fractions of the recombinant *E. coli* cells harboring pTrcMV^140^PL on the Coomassie Blue-stained SDS-PAGE gel (Fig. 3A). Additionally, the amount and localization of MV^140^-fused lipase on membrane fractions were clearly confirmed by western blotting (Fig. 3B). These results show that the fusion lipase tagged with FLAG was successfully displayed on the *E. coli* surface. However, the localization of excess proteins on the outer membrane might cause problems in cell wall integrity and could consequently result in cell lysis and the possible release of anchored proteins into the culture medium. Thus, the culture supernatant of *E. coli* harboring pTrcMV^140^PL was analyzed by western blotting analysis, but no signal was detected. These results indicate that MV^140^-fused lipases were displayed on the surface with a minimum of cell lysis.

### Confocal Microscopic Analysis of Lipase Display on the *E. coli* Surface

**Fig. 4:**
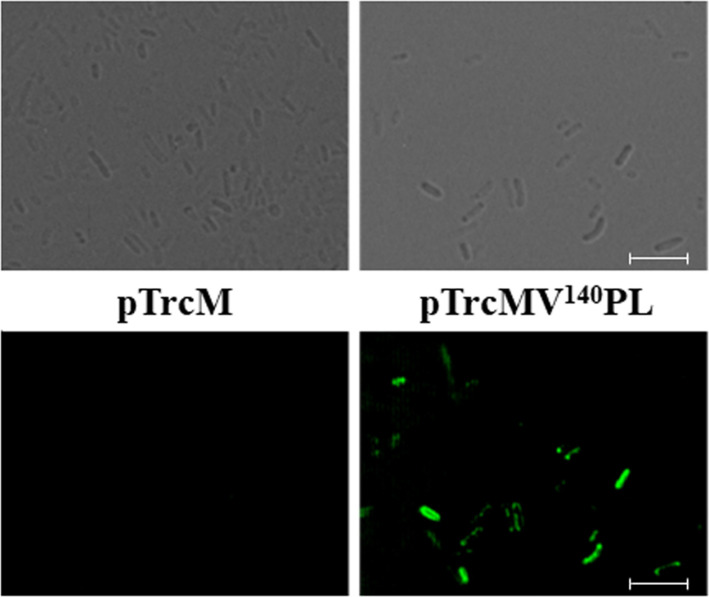
Differential interference microscope images (upper) and confocal immunofluorescence microscope images (lower) of *E. coli* XL10-Gold cells harboring pTrcM (control; left images) and pTrcMV^140^PL (right images). Each scale bar represents 5 μm.

Additionally, the localization of lipase was confirmed by confocal microscopy. After cultivation, the cells were labeled with FITC-conjugated anti-FLAG antibody probe, which can recognize the FLAG tag linked to the C- terminus of lipase. *E. coli* harboring pTrcMV^140^PL showed strong fluorescence, while *E. coli* harboring pTrcM did not show any fluorescence signal (Fig. 4). This finding means that the MV^140^ anchoring motif successfully mediated the localization of lipase on the surface of *E. coli*. This result is almost consistent with the results of enzyme activity and western blotting of the MV^140^-fused lipase on the outer membrane of *E. coli* (Figs. 2 and 3).

### Display of α-Amylase on the *E. coli* Surface Using the MV^140^ as an Anchoring Motif

**Fig. 5:**
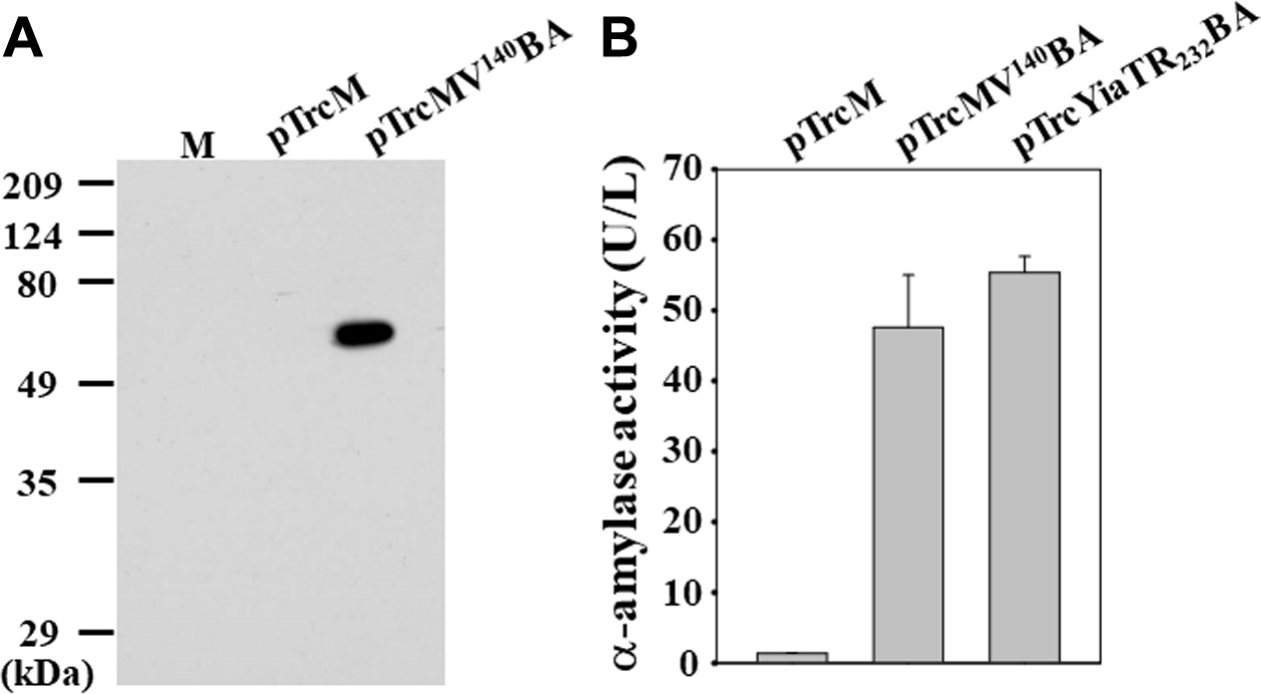
Display of *B. subtilis* α-amylase on the *E. coli* cell surface. (**A**) Western blotting analyses of the outer membrane fractions of *E. coli* XL10-Gold cells harboring pTrcM (control) or pTrcMV^140^BA. M indicates the molecular weight size markers (kDa). (**B**) The α-amylase activity of *E. coli* XL10-Gold cells harboring pTrcM (control), pTrcMV^140^BA or pTrcYiaTR_232_BA [20].

To demonstrate the general use of MV^140^ motif, *B. subtilis* α-amylase was examined as another model protein for cell surface display. First, the *B. subtilis* α-amylase gene containing the FLAG sequence was obtained from pTrcYiaTR_232_BA [20] with digestion of the XbaI and HindIII and then cloned into the same sites of the pTrcMV^140^ vector to make pTrcMV^140^BA (Table 1). Confirmation of the display of α-amylase was conducted by analyzing the western blotting of the outer membrane proteins of *E. coli* XL10-Gold cells harboring pTrcMV^140^BA. The result showed that approximately 63-kDa fusion proteins were detected in the outer membrane fractions of *E. coli* XL10- Gold (pTrcMV^140^BA) (Fig. 5A). However, no signal was detected in the *E. coli* cells harboring the control pTrcM.

Next, we examined whether the displayed α-amylase proteins were active. The specific whole-cell α-amylase activities of the recombinant *E. coli* cells are shown in Fig. 5B. As expected, the specific activity of α-amylase in the *E. coli* cells harboring the control pTrcM was not measured. Also, the α-amylase activities were negligible in the supernatants of all recombinant cells, suggesting that cell lysis was not a significant problem. The specific activity of α-amylase with whole cells was 48 U/L for *E. coli* XL10-Gold (pTrcMV^140^BA) cells (Fig. 5B). These results indicate that the MV^140^ motif could be used for successfully displaying another enzyme, α-amylase tagged with FLAG on the *E. coli* cell surface, but the previous motif, YiaTR232 (55 U/L), was a little more efficient as a display motif for α-amylase than the MV^140^. It demonstrates that the display efficiency could be dependent on the proteins of interest for display although it was used with the same anchoring motif. Therefore, this study shows that MipA can be used as a novel anchoring motif for efficient bacterial surface display in the biotechnological and industrial fields.

In summary, a new cell surface display system was developed using the *E. coli* MipA protein in this work. To select the best anchoring site from MipA, six possible sites were tested by designing and constructing the lipase fusion display systems. Among these sites, the enzyme activities showed that MV^140^ was the best anchoring motif for the *E. coli* display system. Further analyses by SDS-PAGE, western blotting, and confocal microscopy suggested that the MV^140^ motif could successfully display highly active forms of lipase on the *E. coli* surface without causing significant defects of cell lysis. In addition, α-amylase was also successfully displayed using the MV^140^-anchoring motif. These two model proteins (lipase and amylase) used in this study could be displayed in active form although they are quite large proteins. However, it might be possible that overexpression of anchoring motifs on the cell surface could result in physiological effects on the rigidity of cell membrane and metabolic burden and then possibly cause cell lysis [1, 30]. To minimize these deleterious effects of surface display, recombinant *E. coli* XL10-Gold was cultured in this study at 30oC after induction, but these mild conditions (*e.g*., low temperatures and lower concentrations of the inducer) result in reduced display of target proteins on the surface and consequently lead to reduced activity [9, 31]. Nevertheless, the *mipA*-truncated fusion display system developed in this work could be used to efficiently display targets (such as peptides, enzymes or proteins) of interest with an active form in biotechnological and industrial applications.

## Figures and Tables

**Table 1:** The bacterial strains and plasmids used in this study.

Strain or Plasmid	Relevant Characteristics	Reference or Source
*E. coli* strains		
XL1-Blue	*recA1 endA1 gyrA96 thi-1 hsdR17 supE44 relA1 lac* [F´ *proAB lacI* ^q^ *Z*Δ*M15* Tn*10* (Tet^r^)]	Stratagene^a^
XL10-Gold	Tet^r^ ∆(*mcrA*)*183* ∆(*mcrCB-hsdSMR-mrr*)*173 endA1 supE44 thi-1 recA1 gyrA96 relA1 lac* Hte [F´ *proAB lacI* ^q^ *Z*∆*M15* Tn*10* (Tet^r^) Amy Cam^r^]	Stratagene^a^
Plasmids		
pTrc99A	4.2 kb; Ap^r^, *trc* promoter	Pharmacia^b^
pTrcM	4.9 kb; pTrc99A derivative containing full-length 747 bp fragment of *E. coli mipA*	This study
pTrcMV^140^	4.6 kb; pTrc99A derivative containing 420 bp fragment of *E. coli mipA*	This study
pTrcMV^176^	4.7 kb; pTrc99A derivative containing 528 bp fragment of *E. coli mipA*	This study
pTrcMK^179^	4.7 kb; pTrc99A derivative containing 537 bp fragment of *E. coli mipA*	This study
pTrcMV^226^	4.9 kb; pTrc99A derivative containing 678 bp fragment of *E. coli mipA*	This study
pTrcMV^232^	4.9 kb; pTrc99A derivative containing 696 bp fragment of *E. coli mipA*	This study
pTrcMK^234^	4.9 kb; pTrc99A derivative containing 702 bp fragment of *E. coli mipA*	This study
pTrcMV^140^PL	6.0 kb; pTrcMV^140^ derivative containing *P. fluorescens* SIK W1 lipase gene fused with FLAG-tag	This study
pTrcMV^176^PL	6.1 kb; pTrcMV^176^ derivative containing *P. fluorescens* SIK W1 lipase gene fused with FLAG-tag	This study
pTrcMK^179^PL	6.1 kb; pTrcMV^179^ derivative containing *P. fluorescens* SIK W1 lipase gene fused with FLAG-tag	This study
pTrcMV^226^PL	6.3 kb; pTrcMV^226^ derivative containing *P. fluorescens* SIK W1 lipase gene fused with FLAG-tag	This study
pTrcMV^232^PL	6.3 kb; pTrcMV^232^ derivative containing *P. fluorescens* SIK W1 lipase gene fused with FLAG-tag	This study
pTrcMK^234^PL	6.3 kb; pTrcMK^234^ derivative containing *P. fluorescens* SIK W1 lipase gene fused with FLAG-tag	This study
pTrcMV^140^BA	5.9 kb; pTrcMV^140^ derivative containing *B. subtilis* α-amylase gene fused with FLAG-tag	This study

a Stratagene Cloning System, USA.

b Pharmacia Biotech, Uppsala, Sweden.

**Table 2:** The list of primers used in the PCR experiments.

Primer no.	Sequence^a^	Gene to be amplified	Template
1	5-g**gaattc**ATGACCAAACTCAAACTTCTGGCA	Full-length *mipA* gene	*E. coli* W3110 chromosome
2	5-gc**tctaga**TCAGAATTTGTAGGTGATCCCGGT		
3	5-g**gaattc**ATGACCAAACTCAAACTTCTGGCA	Truncated *mipA* at Val^140^ position	*E. coli* W3110 chromosome
4	5-gc**tctaga**GACGATGCCGTTGCTGTTATCC		
5	5-g**gaattc**ATGACCAAACTCAAACTTCTGGCA	Truncated *mipA* at Val^176^ position	*E. coli* W3110 chromosome
6	5-gc**tctaga**TACGCCATAATAGTATTCGTTCTG		
7	5-g**gaattc**ATGACCAAACTCAAACTTCTGGCA	Truncated *mipA* at Lys^179^ position	*E. coli* W3110 chromosome
8	5-gc**tctaga**TTTGCGCGATACGCCATAATAGTA		
9	5-g**gaattc**ATGACCAAACTCAAACTTCTGGCA	Truncated *mipA* at Val^226^ position	*E. coli* W3110 chromosome
10	5-gc**tctaga**AACTTCATCAGACAGACGGGTGTA		
11	5-g**gaattc**ATGACCAAACTCAAACTTCTGGCA	Truncated *mipA* at Val^232^ position	*E. coli* W3110 chromosome
12	5-gc**tctaga**CACCATCGGGCTGTCAGTAACTTC		
13	5-g**gaattc**ATGACCAAACTCAAACTTCTGGCA	Truncated *mipA* at Lys^234^ position	*E. coli* W3110 chromosome
14	5-gc**tctaga**TTTATCCACCATCGGGCTGTCAGT		
15	5-gc**tctaga**ATGGGTGTATTTGACTACAAGAAC	*P. fluorescens* SIK W1 lipase gene fused with FLAG tag	*P. fluorescens* SIK W1 chromosome [28,29]
16	5-ccc**aagctt**ttacttgtcgtcatcgtccttgtagtcACTGATCAGCACACC		

a Restriction enzyme sites are show

*tliA*, which encodes a thermostable lipase (476 aa; 49.9 kDa).
